# Knowledge, risk perception and uptake of COVID-19 vaccination among internally displaced persons in complex humanitarian emergency setting, Northeast Nigeria

**DOI:** 10.1186/s12889-024-18164-y

**Published:** 2024-02-28

**Authors:** Saheed Gidado, Melton Musa, Ahmed Ibrahim Ba’aba, Mark Rohit Francis, Lilian Akudo Okeke, Fatima Lawan Bukar, Patrick M. Nguku, Idris Suleman Hadejia, Isa Ali Hassan, Ibrahim Muhammad Bande, Martins Onuoha, Rabi Usman, Gideon Ugbenyo, Ntadom Godwin, Elsie Ilori, Aisha Aliyu Abulfathi, Lawi Auta Mshelia, Abede Momoh Mohammed, Muhammad Maijawa Abdullahi, Mohammed Isa Bammami, Pekka Nuorti, Salla Atkins

**Affiliations:** 1 Health Sciences Unit, Faculty of Social Sciences, Tampere University, Tampere, Finland; 2 African Field Epidemiology Network, Borno State Field Office, Maiduguri, Nigeria; 3 African Field Epidemiology Network, Yobe State Field Office, Damaturu, Nigeria; 4 African Field Epidemiology Network, Adamawa State Field Office, Yola, Nigeria; 5 Department of Community Medicine, University of Maiduguri, Maiduguri, Borno State Nigeria; 6 African Field Epidemiology Network, Nigeria Country Office, Abuja, Nigeria; 7 Department of Community Medicine, Ahmadu Bello University, Zaria, Kaduna State Nigeria; 8 Borno State Ministry of Health, Maiduguri, Borno State Nigeria; 9 Department of Disease Control and Immunization, Yobe State Primary Health Care Board, Damaturu, Yobe State Nigeria; 10 Nigerian Correctional Service, Adamawa State Office, Yola, Adamawa State Nigeria; 11 Resolve to Save Lives, Abuja, Nigeria; 12 Epidemiology Division, Federal Ministry of Health, Abuja, Nigeria; 13 Nigeria Centre for Disease Control and Prevention, Abuja, Nigeria; 14 Borno State Primary Health Care Development Agency, Maiduguri, Borno State Nigeria; 15 World Health Organization, Borno State Office, Maiduguri, Nigeria; 16 Nigeria Field Epidemiology and Laboratory Training Program, Abuja, Nigeria; 17 Yobe State Hospitals Management Board, Damaturu, Yobe State Nigeria

**Keywords:** Internally displaced persons, COVID-19, COVID-19 vaccines, Perception, Cross-sectional study, Nigeria

## Abstract

**Background:**

Owing to crowded and unsanitary conditions, internally displaced persons (IDPs) have an increased risk of COVID-19 infection. Adoption of COVID-19 preventive measures among this population is premised on accurate information, adequate knowledge, and risk perception. We assessed COVID-19 knowledge and risk perception and investigated the association between risk perception and COVID-19 preventive measures, including vaccination among IDPs in Northeast Nigeria.

**Methods:**

We conducted a cross-sectional study during July–December 2022 and sampled 2,175 IDPs using stratified sampling. We utilized a 12-point assessment tool to evaluate COVID-19 knowledge. Participants who scored ≥ 6 points were considered to have adequate knowledge. We used a 30-item Risk Behavior Diagnosis Scale to assess COVID-19 risk perception and evaluated each item on a 5-point Likert scale. Participants were divided into risk perception categories by the median of Likert scale scores. We performed weighted logistic regression analysis to identify factors associated with risk perception. Pearson’s chi-squared with Rao-Scott adjustment was used to determine the relationship between risk perception and COVID-19 preventive measures.

**Results:**

Of 2,175 participants, 55.7% were 18–39 years old, 70.9% were females, and 81.7% had no formal education. Among the IDPs, 32.0% (95% CI: 28.8 – 35.0) were considered to have adequate COVID-19 knowledge, and 51.3% (95% CI: 47.8 – 54.8) perceived COVID-19 risk as high. Moreover, 46.3% (95% CI: 42.8 – 50.0) had received one dose of COVID-19 vaccine, and 33.1% (95% CI: 29.8 – 36.0) received two doses. Adequate knowledge (Adjusted Odds Ratio (AOR) = 2.10, [95% CI: 1.46 – 3.03]) and post-primary education (AOR = 3.20, [95% CI: 1.59 – 6.46]) were associated with risk perception. Furthermore, high risk perception was significantly associated with wearing face masks (χ^2^ = 106.32, *p*-value < .001), practicing hand hygiene (χ^2^ = 162.24, *p*-value < .001), physical distancing (χ^2^ = 60.84, *p*-value < .001) and vaccination uptake (χ^2^ = 46.85, *p*-value < .001).

**Conclusions:**

This study revealed gaps in COVID-19 knowledge, risk perception, and vaccination uptake but demonstrated a significant relationship between risk perception and COVID-19 preventive practices. Health education and risk communication should be intensified to improve knowledge, elicit stronger risk perception, and enhance COVID-19 preventive practices.

**Supplementary Information:**

The online version contains supplementary material available at 10.1186/s12889-024-18164-y.

## Background

Coronavirus disease (COVID-19), first reported in Wuhan, China, in December 2019, has evolved from a local outbreak to a global pandemic [1]. Besides causing considerable morbidities and mortalities, COVID-19 has negatively impacted health systems worldwide. The pandemic has disrupted healthcare delivery services, including malaria, HIV, Tuberculosis, and immunization services, particularly in Sub-Saharan Africa (SSA) [2, 3]. Following the confirmation of the first COVID-19 case in Nigeria on 27 February 2020, the Government, through the Federal Ministry of Health, activated a multi-sectoral national COVID-19 Emergency Operations Centre (EOC). The country enhanced surveillance at facility and community levels, expanded laboratories for COVID-19 testing, and intensified risk communication activities, among other measures [4, 5]. Further, the Nigerian Government flagged off a COVID-19 vaccination campaign on 5 March 2021. As of 30 January 2023, about 67% of the country’s nearly 116 million eligible population had received at least one dose of COVID-19 vaccine [6].

Over the years, the world has witnessed a rising trend of humanitarian crises. As of the end of 2021, there were unprecedented 59.1 million internally displaced persons (IDPs) globally, 46% of whom were in SSA [7]. Sadly, these crisis-affected population are disproportionately affected by the health and socio-economic impact of COVID-19 pandemic [8]. Among the countries in SSA, Nigeria has the fourth largest population of IDPs after the Democratic Republic of Congo (DRC), Ethiopia, and South Sudan [7]. Since 2009 when Non-State Armed Groups (NSAG) started an armed rebellion against the Nigerian Government, Borno, Adamawa, and Yobe States in the country’s northeast region have witnessed immense armed conflicts and generalized violence [9]. The chronic insurgency triggered a complex humanitarian emergency in the region with extensive destruction of lives and properties. Due to the protracted humanitarian crisis, over two million IDPs, predominantly women and children, have been displaced in the region [10].

Typically, IDPs are displaced to camps, makeshift structures, and other camp-like settings, often characterized by overcrowding, poor water, sanitation, and hygiene (WASH), and reduced food security [11, 12]. Owing to their congested and unsanitary living conditions, IDPs possess an elevated risk of contracting and transmitting infectious diseases, including COVID-19, with potentially fatal consequences [12, 13]. Therefore, adoption of recommended COVID-19 preventive measures including vaccination, is imperative to limit COVID-19 transmission among this population. However, adoption of these measures at individual and household levels is premised largely on accurate information, adequate knowledge, and risk perception. According to the World Health Organization (WHO), the best approach to prevent and reduce COVID-19 transmission is to be well-informed and knowledgeable about the disease [14]. Similarly, existing evidence indicates that disease risk perception is crucial in motivating behavioral health changes necessary to limit disease transmission and accelerate control [15]. In view of their high risk status, assessment of COVID-19 knowledge and risk perception among IDPs is essential to identify gaps and inform appropriate interventions to enhance COVID-19 preventive and control measures among this population.

Generally, there is a paucity of research in the published literature on COVID-19 knowledge and risk perception among IDPs in humanitarian context. Few studies conducted in DRC, Somalia, and Sudan to assess COVID-19 knowledge among IDPs reported widely contrasting findings [16–18]. In Nigeria, there is hardly any published study that assessed COVID-19-related knowledge among IDPs in complex emergency situation. Moreover, several studies have explored COVID-19 risk perception among the general population [19–21]. However, there is a dearth of similar research conducted among IDPs in humanitarian situations. Furthermore, as part of the global response to the widespread misconceptions and conspiracy theories regarding COVID-19 vaccine safety and effectiveness, many studies were commissioned to investigate the impact of these controversies on vaccine acceptance and uptake among the general population [22–25]. Unfortunately, there is a lack of studies assessing COVID-19 vaccination uptake among IDPs in humanitarian emergencies. As previously narrated by several authors, findings of research conducted among the general population in stable, non-humanitarian contexts cannot simply be extrapolated to inform disease prevention and control interventions in humanitarian emergencies [26].

Given the transmission potential of COVID-19 among IDPs in congested settings, the programmatic necessity to enhance COVID-19 pandemic response in this context, and the dearth of research on this subject in the published literature, we implemented this field-based, collaborative research. We aimed to determine COVID-19 knowledge and risk perception, identify factors associated with COVID-19 risk perception, and ascertain the association between risk perception and COVID-19 preventive practices, including vaccination uptake among IDPs in northeast Nigeria.

### Theoretical framework

This study is theoretically grounded in the Extended Parallel Process Model (EPPM). EPPM is a fear appeal theory developed by a communication expert, Kim Witte, in 1992 [27]. Essentially, EPPM provides a theoretical framework that illustrates how people appraise health risk and describes their subsequent response to the risk based on the risk appraisal [28]. The EPPM posits that a health risk typically induces two cognitive appraisals in affected individuals – an appraisal of threat (perceived threat) and an appraisal of efficacy of the recommended response (perceived efficacy) [27].

Conceptually, perceived threat is defined as a cognition about a danger or harm that exists in an individual’s environment [29]. Perceived threat comprises two dimensions, namely 1) perceived susceptibility and 2) perceived severity. Whereas perceived susceptibility signifies the beliefs about one’s risk of experiencing a threat, perceived severity indicates the beliefs about the significance or magnitude of the threat, and the beliefs relating to the consequences should a given event occur. Moreover, perceived efficacy implies cognitions about effectiveness, feasibility, and ease with which a recommended response hinders or averts a threat [29]. Perceived efficacy also contains two underlying dimensions, which are 1) perceived self-efficacy and 2) perceived response efficacy. Perceived self-efficacy connotes beliefs about one’s ability to perform the recommended response to avert the threat. In contrast, perceived response efficacy is the belief about the effectiveness of recommended response in deterring or preventing the threat [29].

According to the EPPM, individuals exhibit different control responses when confronted with a health risk. These control responses are determined by individuals’ cognitive appraisal of the threat and the efficacy associated with the health risk [28, 29]. Table 1 shows the interaction of perceived threat and perceived efficacy to produce the different EPPM control responses.

**Table 1:** Interaction of perceived threat and perceived efficacy to produce EPPM^a^ control responses

S/N	Perceived threat	Perceived efficacy	Type of control responses	Attitudinal dispositions and description
1	High	High	Danger control response	**Responsive** – Individuals are well motivated to undertake preventive and control measures to protect themselves against health risk. Usually manifests in positive changes in attitude, intention, and behavior.
2	High	Low	Fear control response	**Avoidant** – Affected individuals doubt their ability to perform the recommended responses and/or doubt the efficacy of such responses. Such individuals engage in defensive motivation using various psychological defense strategies, including defensive avoidance, denial, and reactance, to control their fears.
3	Low	High	Lesser danger control response	**Proactive** – Individuals taking some protective actions but are not really motivated to do much.
4	Low	Low	No control response	**Indifferent** – Individuals not considering the risk to be real or relevant to them; often not even aware of the risk. Affected individuals do not undertake any measure to protect themselves against health risk.

^a^Extended Parallel Process Model

In this study, we applied the EPPM theoretical framework to conduct a structured, comprehensive assessment of the constructs of COVID-19 risk perception (perceived COVID-19 susceptibility, severity, self-efficacy, and response efficacy) based on the EPPM definitions and explanations of these constructs, as presented earlier. The EPPM also afforded a well-conceptualized approach to evaluate COVID-19 risk perception, identify factors associated with risk perception and determine the relationship between risk perception and COVID-19 preventive measures. Through the practical application of the EPPM framework, we were able to categorize the participants into risk control responses and describe their attitudinal dispositions to COVID-19 risk.

## Methods

### Study design

This cross-sectional study was conducted among IDPs in selected IDPs camps in Borno, Adamawa, and Yobe States (BAY States), between July and December 2022.

### Study setting

Borno, Adamawa, and Yobe States are among Nigeria’s 36 States (and Federal Capital Territory). The three states are situated in the northeastern part of the country, sharing international borders with Niger, Chad, and Cameroun. Borno State has an estimated population of 6,629,190, Adamawa has 4,727,312 while Yobe has 3,757,947 [30]. These three states host about 284 IDPs camps and camp-like settings, with an estimated 195,901 households and 855,020 IDPs [31]. Whereas a number of these IDPs camps are designated as official (formal) camps because of the presence of Government authorities and camp management structure, several others are unofficial (informal) camps. School attendance among the children in this setting is very low. For instance, of the 284 camps/camp-like settings, only 2% had more than 75% of children attending school [31]. Health care services are provided by facilities in IDPs camps, particularly the formal camps. Several other health facilities outside the camps, but mostly within the host communities, also serve the IDPs.

### Target population, study population and study participants

The target population for this study were IDPs residing in IDPs camps in Borno, Adamawa, and Yobe States. The study population were IDPs living in selected (study) IDPs camps in these States during the study period. Study participants were individuals aged 18 years and above sampled from among the study population.

### Sample size

The sample size for the study was determined using the approach recommended by Lwanga and Lemeshow, and reported by other authors [32, 33]. To compute the sample size, we assumed an anticipated population proportion (proportion of IDPs with sufficient knowledge regarding COVID-19) reported as 15% by Claude et al. among IDPs in DRC [16]. Furthermore, we assumed a confidence level of 95%, and a margin of error of 4%. The effective sample size of 306 participants per state was inflated by a factor of 2, our assumed design effect, to account for selecting study participants via a sampling method other than simple random sampling [34, 35]. The actual sample size of 612 per state was adjusted to 680 per state to account for a possible 10% non-response rate. Across the three states, we determined a minimum sample size of 2,040 participants.

### Selection of study IDPs camps

For practical and logistic considerations, we selected 18 IDPs camps across the three states – six in each state. To select the study camps, we utilized the International Organization for Migration (IOM), Displacement Tracking Matrix (DTM) – Northeast Nigeria Displacement Report Round 40 (March 2022) [31]. Among other characteristics, IOM displacement reports document displaced population estimates at household and individual levels. From the displacement report, we adopted key criteria to guide the selection of study camps. These criteria included: 1) population size of the camps, with considerations for large camps that offer a reasonable representation of IDPs from different districts and tribes, 2) status of IDPs camp (whether formal or informal), 3) geographical spread of camps across different districts, 4) a mixture of camps supported by different organizations, and 5) camps with minimal security risk to the research team. The sample size for each state was distributed equally among the selected (study) IDPs camps in the respective states.

### Selection of households and respondents

For the purpose of this study, we defined a household as a group of people who eats from the same pot. We employed a stratified random sampling approach to select households and respondents. This method is one of the recommended approaches for selecting health research participants in displacement contexts [36]. We stratified each IDPs camp into four distinct, well-delineated geographical strata. This stratification process leveraged the polio eradication program’s house-to-house vaccination teams’ microplanning approach [37]. To determine the required number of households in each stratum, we distributed the camp sample size across the four strata in the respective camps, proportionate to the population size of each stratum. To accomplish this proportionate allocation, we applied the formular: #HHs = [(Sp/Cp)*Css]; where #HHs = Required number of households in each stratum, Sp = Stratum population, Cp = Camp population and Css = Camp sample size. We employed a simple random sampling technique to select households in each stratum. To facilitate the selection of households, we utilized the polio eradication program’s microplanning enumerated household listing data.^1^ In each selected household, one household member aged 18 years or older was selected using a simple random sampling technique, and then interviewed.

### Data collection instrument and study variables

We collected data using a semi-structured data collection instrument. The data instrument comprised 62 items categorized under six major sections, namely 1) socio-demographic characteristics, 2) IDP camp characteristics, 3) COVID-19-related knowledge, 4) COVID-19 risk perception, 5) preventive practices regarding COVID-19, and 6) uptake of COVID-19 vaccination. The items in the instrument were informed by a review of the literature on subjects similar to our research. These items conform with WHO guidelines and recommendations for COVID-19 prevention and control [38, 39]. Additionally, the items were appropriately contextualized to reflect the educational level of the study participants and the peculiarities of the research setting. The instrument was developed in English language and translated into the local language (Hausa) prior to data collection. The section on socio-demographic characteristics captured data on age, gender, educational level, marital status, religion, occupation, and average monthly household income. Similarly, the IDPs camp characteristics section captured data on the status of the camps (formal or informal), year of camp establishment, and presence of health facilities in the camps, among others.

The section on COVID-related knowledge included questions on the cause of COVID-19, signs and symptoms of COVID-19, mode of spread, and COVID-19 preventive and control measures. Furthermore, the data instrument had a list of 30 items to assess participants’ COVID-19 risk perception across the four dimensions of severity, susceptibility, self-efficacy, and response efficacy according to the theoretical framework of EPPM. The study instrument was also populated with items that inquired about participants’ COVID-19 preventive practices. The final section of the instrument included questions on the uptake of COVID-19 vaccination, the number of vaccine doses received (based on history and vaccination card), and the reasons for non-vaccination as indicated. The instrument was field-tested in three non-study camps across the three states to assess the appropriateness of the study items and establish the content validity of the data tool.

### Data collection

Data were collected by locally sourced, well-trained interviewers. These interviewers conducted face-to-face interviews with the study participants to collect data electronically using open data kit (ODK) – an open-source mobile data collection platform uploaded on internet enabled Android devices. This platform facilitated data submission to the backend server immediately after every interview. The quality of data collection was further enhanced by field monitoring and supervision, as well as real time data quality checks from the backend server. Importantly, the interviewers and field supervisors adhered to recommended COVID-19 preventive and control measures, including the use of facemasks and physical distancing during data collection.

### Assessment of COVID-19 knowledge

We used a 12-point assessment tool to determine participants’ COVID-19 knowledge across three domains: 1) signs and symptoms, 2) mode of spread, and 3) preventive measures [40, 41]. One point was recorded for every correct response. Participants who scored 6 points and above were considered to have adequate knowledge, while those who scored less than 6 points possessed poor knowledge [42]. The items employed to assess COVID-19 knowledge are presented in the Supplementary file 1 (S1).

### Assessment of COVID-19 risk perception

We adapted the Risk Behavior Diagnosis (RBD) scale to assess participants’ COVID-19 risk perception. Firmly rooted in the EPPM, the RBD scale is a 12-item scale that assesses risk perception across four dimensions, namely perceptions of susceptibility, severity, self-efficacy, and response efficacy [43]. The original RBD scale assesses each of these dimensions with three items on a 7-point Likert scale ranging from 1 (strongly disagree) to 7 (strongly agree). However, for our study, we increased the number of items from 12 to 30. We assessed perceived susceptibility and severity with three items each. In contrast, we utilized 12 items apiece to assess perceived self-efficacy and response efficacy. This was necessary to accommodate the recommended COVID-19 preventive measures, including the use of face masks, hand hygiene practices, physical distancing, and COVID-19 vaccination. We used Cronbach’s alpha coefficient of reliability to measure the internal consistency of the four dimensions (scales). The Cronbach’s alpha values were 0.93, 0.92, 0.95, and 0.95 for perceived susceptibility, severity, self-efficacy, and response efficacy scales respectively, indicating very high internal consistency of these scales.

Furthermore, for each item across all the four dimensions, we collapsed the 7-point Likert scale to 5 points, ranging from 1 (strongly disagree) to 5 (strongly agree) to reduce confusion in data collection and improve response quality [19]. We determined the composite scores for perception of threat by adding up the Likert scale scores for perceived susceptibility and severity. Likewise, we summed the scores for perceived self-efficacy and response efficacy to obtain the composite scores for perception of efficacy. The Likert scale scores for all four dimensions were added up to obtain the overall risk perception scores.

For standardization and comparison, the scores were rescaled to 0 – 100 [44, 45]. To rescale the scores, we used the formula:

$$\text{Y}=\frac{\left(\text{X}-\text{X}min\right)\mathrm n}{\text{X}range}$$

Where Y is the new adjusted value, X is the original value, X*min* is the minimum value on the original scale, X*range* is the difference between the highest and lowest value on the original scale, and n is the upper limit of the rescaled value, which in our study was 100. We then performed a median split on the rescaled scores to dichotomize perception of threat (low and high threat) and perception of efficacy (low and high efficacy), consistent with approaches reported in previous literature [46, 47]. This dual dichotomization categorized study participants into four groups as follows: 1) high threat and high efficacy, 2) high threat and low efficacy, 3) low threat and high efficacy, and 4) low threat and low efficacy [29]. As presented in Table 1, participants in each group exhibit different EPPM control responses. Additionally, a median split of the rescaled overall risk perception score dichotomized participants into low risk perception and high risk perception categories. The 30 items used to assess COVID-19 risk perception are presented in the Supplementary file 2 (S2).

### Data analysis and statistical methods

We performed complex sample survey data analysis to account for the differential probabilities of selecting study participants due to the complex sampling approach. We employed an inverse-probability weighting approach to obtain participants’ survey weights. We incorporated these survey weights in the complex survey data analysis and computed weighted statistical estimates, standard errors, and confidence intervals. Univariate analysis was conducted to describe participants’ socio-demographic and other characteristics. We used Pearson’s chi-squared test with second-order Rao-Scott adjustment (F-distribution) to examine the relationship between categorical variables, consistent with recommended approach for complex sample survey analysis [48]. Further, we performed weighted binary logistic regression analysis to identify factors associated with COVID-19 risk perception. We constructed weighted crude logistic regression models to determine unadjusted association between each explanatory variable and COVID-19 risk perception, designating low COVID-19 risk perception as the outcome reference category. Thereafter, we employed a backward elimination model-building approach to construct weighted multivariable logistic regression models. We obtained weighted adjusted odds ratio and 95% confidence interval for the explanatory variables. We used the variance inflation factor (VIF) to assess multicollinearity among the explanatory variables in the multivariable models. The VIF values for all the variables were less than 5, indicating low multicollinearity among these variables. Finally, we employed Akaike Information Criterion (AIC) to compare the quality of the different model candidates and selected the model with the smallest AIC value as the most parsimonious for our data. Data were analyzed using R statistical and computing software version R-4.2.2.

### Reporting

This research was reported based on the Guidelines for Reporting Observational Studies, in accordance with the Strengthening the Reporting of Observational Studies in Epidemiology (STROBE) Statement [49].

## Results

### Socio-demographic characteristics of respondents

A total of 2,175 sampled IDPs participated in the study; all were interviewed. The weighted median age of the IDPs was 36 years (95% CI: 35 – 38 years), with an interquartile range of 15 years. Among the IDPs, 23.3% (95% CI: 20.4 – 26.0) were 18 to 29 years old, while 32.4% (95% CI: 29.1 – 36.0) were 30 to 39 years old. Most were females – 70.9% (95% CI: 67.7 – 74.0) and had no formal education – 81.7% (79.0 – 84.0). Further, 47.6% (95% CI: 44.2 – 51.0) were unemployed while 66.4% (95% CI: 63.1 – 70.0) had lived in IDPs camps for more than five years. The socio-demographic characteristics of the IDPs are presented in Table 2.

**Table 2:** Socio-demographic characteristics of respondents

Characteristics (*N* = 2175)	Number of respondents	Weighted % (95% CI)
**Age group (years)**		
18—29	626	23.3 (20.4—26.0)
30—39	695	32.4 (29.1—36.0)
40—49	480	27.1 (24.0—30.0)
≥ 50	374	17.2 (14.5—20.0)
**Sex**		
Male	766	29.1 (25.9—32.0)
Female	1409	70.9 (67.7—74.0)
**Highest level of formal education attained**		
None	1530	81.7 (79.0—84.0)
Primary	369	9.8 (7.8—12.0)
Post primary (secondary, tertiary)	276	8.5 (6.6—10.0)
**Marital Status**		
Never married	176	6.8 (5.0—9.0)
Presently married	1730	74.4 (71.3—77.0)
Widowed	154	9.0 (7.0—11.0)
Others^a^	115	9.8 (7.7—12.0)
**Religion**		
Christianity	109	1.0 (0.4 -2.0)
Islam	2066	99.0 (98.4—100.0)
**Occupation**		
Unemployed	896	47.6 (44.2—51.0)
Traders/Business	356	25.4 (22.4—29.0)
Farmers	742	20.9 (18.1—24.0)
Artisan (skilled laborer)	74	1.8 (0.9—3.0)
Students	57	1.1 (0.4—2.0)
Others^b^	50	3.1 (1.9—4.0)
**Monthly household income**		
< 13,300 NGN^c^	1498	82.3 (79.6—85.0)
≥ 13,300 NGN	677	17.7 (15.1—20.0)
**Duration of residence in IDPs camp**		
≤ 5 years	654	33.6 (30.3—37.0)
> 5 years	1521	66.4 (63.1—70.0)

^a^Separated, divorced, cohabiting

^b^Civil servants, unskilled laborers, drivers

^c^Nigerian Naira (13,300 NGN = 30 US Dollars)

### Respondents’ COVID-19-related knowledge

Table 3 shows IDPs’ COVID-19-related knowledge across various domains. Among them, 45.0% (95% CI: 41.5 – 48.5) knew that COVID-19 is caused by a microorganism. Moreover, 65.9% (95% CI: 62.6 – 69.2) and 59.1% (95% CI: 55.7 – 62.5) knew that cough and fever respectively are symptoms of COVID-19. Additionally, 68.8% (95% CI: 65.6 – 72.0) knew that COVID-19 is spread via close contact, while 50.9% (95% CI: 47.4 – 54.0) knew that physical distancing is a protective measure against COVID-19. Overall, 32.0% (95% CI: 28.8 – 35.0) of the IDPs were considered to have adequate COVID-19-related knowledge.

**Table 3:** Respondents’ COVID-19-related knowledge across key domains

Knowledge domains and questions	Number of respondents	Weighted % (95% CI)
**Causes of COVID-19**		
Microorganism	934	45.0 (41.5—48.5)
Jinn, witchcraft, and other spiritual afflictions	74	7.7 (5.8—9.7)
Cold weather	52	3.7 (2.3—5.0)
Mosquito	33	2.6 (1.4—3.7)
Others^a^	46	2.5 (1.4—3.6)
Poor sanitation	71	0.4 (0.1—0.6)
Don’t know	965	38.2 (34.8—41.6)
**Signs and symptoms of COVID-19**^**b**^		
Cough	1527	65.9 (62.6—69.2)
Fever	1218	59.1 (55.7—62.5)
Headache	692	43.3 (39.8—46.8)
Catarrh	744	17.3 (14.7—19.8)
Difficulty in breathing	556	13.7 (11.4—16.1)
Vomiting	143	5.9 (4.2—7.5)
Joint pain	90	5.8 (4.2—7.5)
Loss of smell	88	4.8 (3.3—6.3)
Diarrhoea	106	4.3 (2.9—5.7)
Tiredness (Fatigue)	68	3.6 (2.3—4.9)
Rash	83	2.9 (1.7—4.0)
Loss of taste	51	2.9 (1.7—4.0)
Don’t know	286	16.2 (13.6—18.8)
**Everyone who gets COVID-19 show signs and symptoms**		
Yes	1211	56.1 (52.7—59.6)
No	204	10.5 (8.3—12.6)
Don’t know	760	33.4 (30.1—36.7)
**Mode of spread of COVID-19**^**b**^		
Through close contact with others	1515	68.8 (65.6—72.0)
Touching contaminated surfaces and objects	694	41.6 (38.1—45.0)
Don’t know	430	21.5 (18.7—24.0)
Drinking polluted water	296	20.8 (18.0—24.0)
Mosquito	109	2.9 (1.8—4.0)
Contact with animal	96	1.7 (0.8—3.0)
**Measures to protect against COVID-19**^**b**^		
Physical distancing	1062	50.9 (47.4—54.0)
Hand hygiene	923	44.3 (40.9—48.0)
Cover mouth and nose while coughing or sneezing	676	32.2 (28.9—35.0)
Avoid crowded places	909	31.1 (27.9—34.0)
Wearing face mask	625	24.1 (21.2—27.0)
COVID-19 vaccination	192	18.0 (15.2—21.0)
Pray	154	15.8 (13.2—18.0)

^a^Act of God, White men, dirty water, rotten fruits, harmattan (dry and dusty wind)

^b^Multiple-response questions

### COVID-19 risk perception with perceived threat and efficacy interaction

Generally, 51.3% (95% CI: 47.8 – 54.8) of the IDPs perceived the risk of COVID-19 to be high. Table 4 shows the IDPs’ COVID-19 risk perception categorized by perceived threat and perceived efficacy. Among the IPDs, 55.0% (95% CI: 51.5 – 58.5) perceived the threat posed by COVID-19 as low, while 48.9% (95% CI: 45.4—52.4) perceived the efficacy of COVID-19 recommended response as low. Less than half of the IDPs – 37.4% (95% CI: 34.0 – 40.7) had low perception of both threat and efficacy.

**Table 4:** COVID-19 risk perception with perceived threat and efficacy interaction

Perceived Threat	Perceived Efficacy				Total	Weighted % (95% CI)
	**High Efficacy**		**Low Efficacy**			
	**Number of respondents**	**Weighted % (95% CI)**	**Number of respondents**	**Weighted % (95% CI)**		
**High Threat**	736	33.4 (30.1—36.7)	189	11.6 (9.3—13.8)	925	45.0 (41.5—48.5)
**Low Threat**	391	17.6 (15.0—20.3)	859	37.4 (34.0—40.7)	1250	55.0 (51.5—58.5)
**Total**	1127	51.1 (47.6—54.6)	1048	48.9 (45.4—52.4)	2175	

### Factors associated with COVID-19 risk perception

The results of weighted crude and multivariable logistic regression analysis of factors associated with COVID-19 risk perceptions are presented in Table 5. Controlling for other covariates, IDPs who had adequate COVID-19-related knowledge compared to those with poor knowledge (Adjusted Odds Ratio (AOR) = 2.10, [95% CI: 1.46 – 3.03]) were significantly more likely to perceive COVID-19 risk as high. Similarly, compared to participants who had no formal education, participants with post-primary education (AOR = 3.20, [95% CI: 1.59 – 6.46]) were significantly more likely to perceive COVID-19 risk as high.

**Table 5:** Results of weighted logistic regression analysis of factors associated with COVID-19 risk perception

Respondents’ characteristics	Univariable model^a^		Multivariable model^a^	
	**COR**^**b**^	**95% CI**	**AOR**^**c**^	**95% CI**
**Knowledge grade**				
Poor (reference)	1.00		1.00	
Good	3.30^*^	2.40—4.55	2.10^*^	1.46—3.03
**Highest educational level attained**				
None (reference)	1.00		1.00	
Primary	2.13^*^	1.32—3.43	1.31	0.77—2.24
Post-primary (secondary, tertiary)	6.37^*^	3.36—12.06	3.20^*^	1.59—6.46
**Presence of health facility in IPDs camp**				
No (reference)	1.00		1.00	
Yes	0.78^*^	0.63—0.97	1.90^*^	1.10—3.28
**Monthly household income**				
< 13,000 NGN^d^ (reference)	1.00		1.00	
≥ 13,000 NGN	2.12^*^	1.46—3.09	2.07^*^	1.37—3.13
**Status of IDPs camp**				
Informal (reference)	1.00		1.00	
Formal	0.30^*^	0.21—0.44	0.41^*^	0.25—0.66
**Marital status**				
Never married (reference)	1.00		1.00	
Presently married	0.19^*^	0.10—0.38	0.24^*^	0.12—0.50
Widowed	0.37^*^	0.16—0.84	0.39^*^	0.16—0.97
Others^e^	0.15^*^	0.07—0.35	0.24^*^	0.10—0.57
**Household distance to nearest health facility**				
< 2 km (reference)	1.00		1.00	
≥ 2 km	0.32^*^	0.22—0.47	0.56^*^	0.37—0.84
**Age group (years)**				
18—29 (reference)	1.00		1.00	
30—39	0.96	0.66—1.40	1.29	0.83—2.01
40—49	0.92	0.62—1.36	1.11	0.68—1.81
≥ 50	0.89	0.57—1.39	0.82	0.46—1.47
**Religion**				
Christianity (reference)	1.00		1.00	
Islam	0.53	0.17—1.62	2.17	0.81—5.80
**Duration of residence in IDPs camp**				
≤ 5 years (reference)	1.00		1.00	
> 5 years	0.95	0.71—1.28	0.78	0.56—1.10
**Sex**				
Male (reference)	1.00		1.00	
Female	0.54^*^	0.39—0.73	0.87	0.60—1.27
**Household size**				
≤ 6 members (reference)	1.00		1.00	
> 6 members	1.12	0.85—1.48	1.25	0.91—1.71

^*^Statistically significant at *P* < 0.05

^a^Low risk perception designated as reference category

^b^Weighted Crude Odds Ratio

^c^Weighted Adjusted Odds Ratio

^d^Nigerian Naira (13,300 NGN = 30 US Dollars)

^e^Separated, divorced, cohabiting

### Uptake of COVID-19 vaccination and reasons for non-vaccination

Table 6 presents the results of COVID-19 vaccination among the IDPs. Based on history (self-reported), 46.3% (95% CI: 42.8 – 50.0) of the IDPs had received at least one dose of COVID-19 vaccine, while 33.1% (95% CI: 29.8 – 36.0) had received at least two doses.

**Table 6:** Uptake of COVID-19 vaccination

COVID-19 vaccination (*N* = 2175)	Number of respondents	Weighted % (95% CI)
**By history**		
At least one dose	638	46.3 (42.8—50.0)
At least two doses	383	33.1 (29.8—36.0)
**By vaccination card**		
At least one dose	568	43.9 (40.4—47.0)
At least two doses	349	30.1 (26.9—33.0)

The reasons given by unvaccinated IDPs for not receiving COVID-19 vaccine are presented in Table 7. COVID-19 myths and misconceptions, concerns about vaccine safety and side-effects, and no felt need were the top reasons for non-vaccination against COVID-19. Additionally, 23% (95% CI: 19.0 – 27.0) of the unvaccinated IDPs refused to provide reasons for not receiving COVID-19 vaccine.

**Table 7:** Reasons for non-vaccination against COVID-19

Reasons for non-vaccination (*N* = 1537)	Number of respondents	Weighted % (95% CI)
Respondents wouldn’t give any reason	310	23.0 (19.0—27.0)
Related to COVID-19 myths and misconceptions	178	19.8 (16.0—23.6)
Concerns about vaccine safety and side effects	309	17.8 (14.2—21.4)
No felt need	213	13.0 (9.9—16.2)
Non-availability of vaccine	308	12.5 (9.4—15.6)
Not around when vaccinators visited	61	7.8 (5.2—10.4)
No consent from husband or household head	38	4.6 (2.5—6.6)
Far distance from household to vaccination post	117	1.4 (0.5—2.2)
Other reasons^a^	48	1.7 (0.5—2.9)

^a^Pregnancy-related, sickness, don’t know where to receive vaccine, vaccination teams did not visit

### Association between risk perception and COVID-19 preventive practices

As shown in Table 8, there was a significant association between COVID-19 risk perception and adoption of COVID-19 preventive measures investigated. Among IDPs who perceived COVID-19 risk as high, 68.0% (95% CI: 63.3 – 72.6) reportedly wear face masks regularly, compared to 31.3% (95% CI: 26.7 – 35.9) of those who perceived the risk as low (χ^2^ (1) = 106.32, *p*-value < 0.001). Similarly, 76.9% (95% CI: 72.8 – 80.9) of IDPs who perceived COVID-19 risk as high reportedly practice hand hygiene compared to 31.9% (95% CI: 27.3 – 36.6) of those who perceived the risk as low (χ^2^ (1) = 162.24, *p*-value < 0.001). Likewise, among IDPs with high perception of COVID-19 risk, 58.2% (95% CI: 53.4 – 62.9) had received at least one dose of COVID-19 vaccine compared to 33.8% (95% CI: 29.0 – 38.5) of those with low perception of risk (χ^2^ (1) = 46.85, *p*-value < 0.001).

**Table 8:** COVID-19 risk perception and adoption of preventive measures

COVID-19 preventive measures	Low risk perception (*N* = 1085)		High risk perception (*N* = 1090)		Chi-squared^a^*, p*-value
	**Number of respondents**	**Weighted % (95% CI)**	**Number of respondents**	**Weighted % (95% CI)**	
**Wear face mask regularly**					
No	585	68.7 (64.1—73.3)	248	32.0 (27.4—36.6)	106.32, < 0.001
Yes	500	31.3 (26.7—35.9)	842	68.0 (63.3—72.6)	
**Practice hand hygiene**					
No	651	68.1 (63.4—72.7)	319	23.1 (19.0—27.2)	162.24, < 0.001
Yes	434	31.9 (27.3—36.6)	771	76.9 (72.8—80.9)	
**Practice physical distancing**					
No	935	86.5 (83.1—89.9)	711	62.1 (57.4—66.9)	60.84, < 0.001
Yes	150	13.5 (10.1—16.9)	379	37.9 (33.1—42.6)	
**Avoid indiscriminate touching of objects and surfaces**					
No	960	86.5 (83.0—89.9)	846	72.6 (68.2—76.9)	22.83, < 0.001
Yes	125	13.5 (10.1—16.9)	244	27.4 (23.0—31.8)	
**Avoid crowd**					
No	934	93.2 (90.8—95.6)	814	86.8 (83.6—90.0)	9.45, 0.002
Yes	151	6.8 (4.4—9.2)	276	13.2 (9.9—16.4)	
**Received at least one dose of COVID-19 vaccine**^**b**^					
No	884	66.2 (61.5—71.0)	653	41.8 (37.0—46.6)	46.85, < 0.001
Yes	201	33.8 (29.0—38.5)	437	58.2 (53.4—62.9)	
**Received two doses of COVID-19 vaccine**^**b**^					
No	980	80.4 (76.4—84.4)	812	54.1 (49.3—59.0)	60.23, < 0.001
Yes	105	19.6 (15.6—23.6)	278	45.9 (41.0—50.7)	

^a^Pearsons’s chi-squared with second-order Rao-Scott adjustment for complex sample survey analysis

^b^Based on history (self-reported)

## Discussion

In this study, we used the EPPM framework to assess COVID-19 risk perception and investigate the association between risk perception and adoption of COVID-19 preventive practices among IDPs in northeast Nigeria. The study aligns with the global efforts to enhance COVID-19 pandemic response, particularly among high-risk groups and contributes to the sparse body of evidence on this subject in the published literature. Most of the IDPs had no formal education, and only about one-third were considered to have adequate COVID-19-related knowledge. More than half of the IDPs perceived the threat posed by COVID-19 to be low, indicating that they perceived COVID-19 susceptibility and/or COVID-19 severity as low. Uptake of COVID-19 vaccination was low; less than half of the IDPs had received at least one dose of the vaccine, while only one-third had received two doses. The study demonstrated a significant association between risk perception and adoption of COVID-19 preventive practices, including vaccination, among this population.

Over 80% of IDPs in this study had no formal education, while approximately 10% were only educated to primary school level. The low level of formal education among this population has implications for disease prevention and control. Generally, individuals with no or low literacy levels are reportedly less likely to utilize disease prevention services [50]. Regarding COVID-19 pandemic, several authors have reported that low level of education is associated with poor adherence to COVID-19 preventive measures [51, 52]. Moreover, prior research have shown that higher education attainment decreases the risk of COVID-19 severity [53]. Similarly, some authors have demonstrated a direct correlation between low education and COVID-19 mortality [54]. A number of studies have also indicated that persons with low education levels are more likely to have COVID-19 misconceptions [55, 56]. Collectively, the above evidence underpins the need for local health authorities to intensify COVID-19 health education and tailored communication to equip the largely uneducated IPDs with the right information necessary to combat misconceptions and inform appropriate actions to limit COVID-19 transmission in this context.

We found considerable COVID-19-related knowledge gaps among the IDPs in our study; only 32% of them possessed adequate COVID-19 knowledge. Compared to studies conducted in a similar context, this figure is higher than the 15% reported in DRC [16], but lower than the 74% obtained in Sudan [57]. The low level of COVID-19 knowledge in the current study could be explained by at least three factors. Firstly, we opine that the low level of COVID-19 knowledge could be due to the low level of formal education among the IDPs. This submission is bolstered by findings of previous studies which demonstrated a positive association between formal education attainment and COVID-19 knowledge [58, 59]. Secondly, displaced population often lack or have limited and infrequent access to timely and reliable information [60]. The restricted access to information in this context could partly, account for the observed gaps in COVID-19 knowledge among the IDPs. A third factor that could explain the relatively low level of COVID-19 knowledge is related to the prevalent erroneous belief in Nigeria that COVID-19 is a hoax and does not exist [61]. It is likely that such misconceptions could spur widespread apathy among the population to learn about the disease, possibly engendering COVID-19 knowledge deficits.

About 55% of the IDPs had a low perception of threat regarding COVID-19 – a proportion higher than the 43% obtained among the general population in Ethiopia [19]. Such IDPs perceived the threat of COVID-19 to be irrelevant or trivial (low perceived susceptibility) and/or insignificant (low perceived severity). Like many other infectious diseases, the transmissibility of COVID-19 is often underestimated, because a large proportion of infected individuals are undetected. This might be due to failure to present at health facilities and/or inadequate testing capacity, as well as the clinical spectrum of the disease [62]. For instance, about 40% of individuals with confirmed COVID-19 infection are asymptomatic [63], while 81% develop only mild symptoms [64]. Therefore, several COVID-19 infected individuals, including those who routinely do not practice any COVID-19 preventive measures, might remain asymptomatic or develop mild illness. This scenario could fuel the wrong assumption that COVID-19 is uncommon, or a mild disease. Additionally, with a pooled case fatality rate of 1% among the general population [65], COVID-19 may be perceived to be a non-lethal disease. Reasonably, these epidemiological descriptions might explain the low perceived COVID-19 susceptibility and severity among a substantial proportion of the IDPs. Given that the cognitive appraisal of health risk typically begins with an appraisal of threat [27], the foregoing interpretation underscores the need for tailored risk communication messages with emphasis on COVID-19 susceptibility and severity to elicit a higher perception of threat among this population.

Despite their increased risk of COVID-19 infection, approximately 37% of IDPs had low perception of both threat and efficacy regarding COVID-19. This figure exceeds the 24% reported in Ethiopia [19]. According to the EPPM, this category of individuals presumably demonstrate an ‘indifferent’ attitudinal disposition and exhibit ‘no control response’. Intuitively, they are unlikely to undertake any preventive and control measures to protect themselves from contracting and transmitting COVID-19. Such individuals potentially fuel disease transmission and undermine COVID-19 pandemic response efforts. We opine that knowledge gaps, dearth of reliable information, COVID-19 misconceptions and distrust of Government could partly explain this undesirable disposition among over one-third of the IDPs. Historically, similar EPPM response characterized the 2013–2014 Ebola virus disease (EVD) outbreaks in Nigeria and other West African countries. Reportedly due to knowledge deficits, information gaps, and widespread misconceptions, several persons did not adopt any recommended preventive and control measures while many others embraced non-scientific measures, including bathing with salt-water solution, and chewing bitter kola (*Garcinia kola*) to protect themselves during these EVD outbreaks [66, 67].

Nearly two years after the Nigerian Government rolled out COVID-19 vaccine in the country, the uptake of COVID-19 vaccination among the IDPs was still low. This finding is worrisome, given that vaccination is one of the key strategies to control the COVID-19 pandemic, particularly among high-risk population. The low COVID-19 vaccination uptake among our study participants could possibly, reflect the general COVID-19 vaccine apathy and poor vaccine acceptance among Nigerians ostensibly, due to widespread misconceptions and misinformation about the vaccine. Uptake of at least one dose of COVID-19 vaccine among the IDPs (46.3% by history, 43.9% by vaccination card) is similar to the findings of other authors who reported an estimated pooled prevalence of 20.0% – 58.2% for COVID-19 vaccination acceptance among Nigerians [68]. In our study, a large number of the unvaccinated IDPs refused COVID-19 vaccine because of myths and misconceptions, vaccine safety and side-effects concerns, and no felt need. Additionally, about one-fifth of the unvaccinated IDPs refused to provide any reason for COVID-19 vaccine rejection – a possible indication of perceived vaccine misconceptions and/or Government mistrust. These findings re-echo the widely reported reasons for COVID-19 vaccine hesitancy documented in the published literature [68–70].

Misconceptions and controversies about vaccine safety and efficacy are not alien to northern Nigeria, where this study was implemented. In 2003, there was a controversy about the safety of the oral polio vaccine (OPV) due to its perceived composition and purported side effects. In several parts of northern Nigeria, OPV was erroneously believed to contain anti-fertility constituents and the human immunodeficiency virus (HIV) [71]. These widespread misconceptions resulted in poor vaccination uptake and a boycott of immunization activities in several states in northern Nigeria [72]. Concerted efforts championed by notable health experts and key religious and traditional leaders in the region reversed the negative impression about OPV and subsequently, improved vaccination uptake. In the light of the experience and lessons learnt from this event, it is expedient for key stakeholders, including experienced health experts, traditional and political leaders, local health authorities, and managers of IDPs camps, to foster strategic partnerships and ramp up health education, social mobilization, and risk communication activities to correct COVID-19 myths and misconceptions and improve COVID-19 vaccination uptake among this population.

We demonstrated a significant relationship between high COVID-19 risk perception and adoption of COVID-19 preventive practices, including vaccination uptake. This finding is consistent with those of previous research which indicate that high disease risk perception motivates preventive behaviour [73, 74]. Although, there are enormous challenges in implementing key COVID-19 preventive measures among the IDPs in humanitarian context [75, 76], it is noteworthy that despite these challenges, high perception of risk inspired the adoption of COVID-19 preventive practices among this population. This finding aligns with the conceptual framework of the Health Belief Model – a socio-psychological model of health behaviour [77]. According to this well-established model, individuals confronted with a health risk are likely to undertake recommended preventive health actions if they perceive the health risk to be high and believe that the perceived benefits of the recommended actions outweigh the perceived barriers [77]. As an essential component of epidemic and pandemic response, this finding highlights the need to intensify risk communication activities among this population to elicit stronger COVID-19 risk perception and motivate the adoption of COVID-19 preventive practices to limit COVID-19 transmission in this context.

This study is anchored on a number of methodological strengths. The application of the EPPM theoretical framework enabled a well-structured, conceptualized assessment of COVID-19 risk perception as well as the interpretation of the findings. Although, this study was conducted among displaced population in humanitarian emergency context, we leveraged available data sources, field-assessment reports, and micro-planning data from notable organizations to enhance the validity of the study. For example, the IOM DTM assessment reports that guided the selection of our study camps are considered among the most comprehensive and reliable displacement reports globally [31]. Furthermore, our study participants were sampled using a stratified random sampling approach. As documented in the literature, we believe this sampling approach possibly, increased the precision of the estimates obtained in our study [35]. Unlike several internet-based studies on similar subject, data for this field-based study were collected via an interviewer-administered questionnaire. This permitted a systematic approach to select study participants, reducing selection bias related to education attainment and internet literacy. Additionally, this strategy enabled us to assess current COVID-19 knowledge and risk perceptions and verify COVID-19 vaccination status recorded on the vaccination cards.

Findings and conclusions from the study should be interpreted within the context of certain limitations. Data on several explanatory and outcome variables were mostly retrospective, self-reported, and not verified. Thus, the potential for recall bias and misinformation cannot be ruled out. Because this was a cross-sectional study, we could not establish temporality and causation for the reported associations. Contextually, we were limited by security concerns in Nigeria’s northeast region and therefore, selected study IDPs camps from locations perceived to be relatively safe and secured at the time of the study.

## Conclusions

Our study revealed poor COVID-19 knowledge, gaps in risk perception and low vaccination uptake among IDPs in northeast Nigeria. However, in spite of the congested humanitarian setting, IDPs who perceived COVID-19 risk as high were more likely to adopt necessary preventive practices, including vaccination uptake to limit COVID-19 transmission. Considering that risk communication is one of the key components of epidemic and pandemic response, our study provides the basis for a robust, responsive, and effective risk communication strategy to induce stronger COVID-19 risk perceptions and inspire the adoption of recommended COVID-19 preventive measures. Importantly, the sub-optimal perception of susceptibility and severity among the participants calls for a review of COVID-19 risk communication messages. Accordingly, the content of COVID-19 risk communication messages should be tailored to emphasize not only perception of efficacy, but also perception of threat to trigger higher perceived susceptibility and severity in this context. Furthermore, local health authorities should synergize with other relevant stakeholders to intensify COVID-19 health education to improve COVID-19 knowledge, correct misconceptions and improve vaccine acceptability. Further qualitative research should explore the roles and impact of social, cultural, and other contextual factors on COVID-19 risk perception and vaccine hesitancy among this population. Finally, we suggest a follow-up study in the near future to reassess the main themes of this study and ascertain progress towards addressing the observed gaps.

### Supplementary Information

**Supplementary Material 1.**

**Supplementary Material 2.**

## Data Availability

This study was conducted in the context of armed conflict induced humanitarian emergency due to the activities of armed insurgent groups against the Nigerian Government. Given the enormous security risk and concerns, the data collected and analyzed from the study are not publicly available. However, the data are available from the corresponding author on reasonable request.

## Abbreviations

AIC: Akaike information criterion
AOR: Adjusted odds ratio
CI: Confidence interval
COVID-19: Coronavirus disease
DTM: Displacement Tracking Matrix
EOC: Emergency Operations Centre
EPPM: Extended parallel process model
EVD: Ebola virus disease
HIV: Human immunodeficiency virus
IDPs: Internally displaced persons
IOM: International Organization for Migration
LGA: Local Government Area
NCDC: Nigeria Centre for Disease Control and prevention
NHREC: National Health Research Ethics Committee of Nigeria
ODK: Open data kit
OPV: Oral polio vaccine
RBD: Risk behaviour diagnosis
SARS-CoV-2: Severe acute respiratory syndrome coronavirus 2
SSA: Sub-Saharan Africa
STROBE: Strengthening the reporting of observation studies in epidemiology
VIF: Variance inflation factor
WASH: Water, sanitation, and hygiene
WHO: World Health Organization

## Footnotes

1 Household listing data are generated through walk-through enumeration activity – an essential component of the immunization program’s micro-planning exercise.
