# Supplementary material for: Knowledge, risk perception and uptake of COVID-19 vaccination among internally displaced persons in complex humanitarian emergency setting, Northeast Nigeria
Source: BMC Public Health. 2024 Feb 28;24:634. doi: 10.1186/s12889-024-18164-y (PMC10902942; doi:10.1186/s12889-024-18164-y)
Supplement: Supplementary file 1 — Supplementary Material 1. [file 12889_2024_18164_MOESM1_ESM.docx]

**Supplementary file (S1): Assessment algorithm for COVID-19-related knowledge**

| **S/N** | **Knowledge domains/questions** | **Anticipated responses** | **Points*** |
| --- | --- | --- | --- |
| 1 | Signs and symptoms of COVID-19?^¶^ | Fever | 1 |
|  |  | Cough | 1 |
|  |  | Fatigue (tiredness) | 1 |
|  |  |  |  |
| 2 | Mode of spread of COVID-19 | Between people who are in close contact (respiratory droplets/short-range aerosol or short-range airborne transmission) | 1 |
|  |  | In poorly ventilated/overcrowded places (long-range aerosol or long-range airborne transmission) | 1 |
|  |  | When people touch their mouth, nose, and eyes after touching surfaces and objects contaminated with the virus | 1 |
|  |  |  |  |
| 3 | Protective measures against COVID-19 | Wearing face mask regularly | 1 |
|  |  | Practicing physical distancing | 1 |
|  |  | Practicing hand hygiene | 1 |
|  |  | Avoiding crowded places | 1 |
|  |  | Covering mouth and nose when coughing or sneezing | 1 |
|  |  | Vaccination with COVID-19 vaccine | 1 |
|  |  |  |  |

* Total of 12 points

^¶^ One point scored for each of the three most common symptoms of COVID-19
