# Supplementary material for: Knowledge, risk perception and uptake of COVID-19 vaccination among internally displaced persons in complex humanitarian emergency setting, Northeast Nigeria
Source: BMC Public Health. 2024 Feb 28;24:634. doi: 10.1186/s12889-024-18164-y (PMC10902942; doi:10.1186/s12889-024-18164-y)
Supplement: Supplementary file 2 — Supplementary Material 2. [file 12889_2024_18164_MOESM2_ESM.docx]

**Supplementary file 2 (S2): List of items* used to assess COVID-19 risk perception across perceived susceptibility, severity, self-efficacy, and response efficacy**

| **Perceived Susceptibility** | |
| --- | --- |
| 1 | I am at risk of getting coronavirus disease (COVID-19) |
| 2 | It is likely (or possible) that I will get coronavirus disease (COVID-19) |
| 3 | I am susceptible to coronavirus disease (COVID-19) |
| **Perceived Severity** | |
| 1 | Coronavirus disease (COVID-19) is a severe disease |
| 2 | Coronavirus disease (COVID-19) can have serious consequences on my life and livelihood |
| 3 | Coronavirus disease (COVID-19) is very harmful |
| **Perceived Self-efficacy** | |
| **Perceived Self-efficacy: Physical distancing** | |
| 1 | It is easy for me to maintain physical distancing to prevent COVID-19 |
| 2 | I can avoid crowded places to reduce my risk of contracting COVID-19 |
| 3 | I am able to practice physical distancing to prevent COVID-19 |
| **Perceived Self-efficacy: Use of face masks** | |
| 4 | I am able to wear face mask regularly to prevent COVID-19 |
| 5 | It is easy for me to wear face mask regularly to prevent COVID-19 |
| 6 | I can wear face mask regularly to prevent COVID-19 |
| **Perceived Self-efficacy: Hand hygiene practices** | |
| 7 | I am able to clean my hands regularly with soap and water or hand sanitizer to prevent COVID-19 |
| 8 | It is convenient for me to clean my hands regularly with soap and water or hand sanitizer to prevent COVID-19 |
| 9 | I can clean my hands regularly with soap and water or hand sanitizer to prevent COVID-19 |
| **Perceived Self-efficacy: COVID-19 vaccination** | |
| 10 | I can take COVID-19 vaccine to protect me from serious illness or death from COVID-19 disease? |
| 11 | I am able to receive COVID-19 vaccine to prevent serious illness or death from COVID-19 disease |
| 12 | It is easy for me to take COVID-19 vaccine to prevent serious illness or death from COVID-19 disease |
| **Perceived Response efficacy** | |
| **Perceived Response efficacy: Physical distancing** | |
| 1 | Maintaining physical distancing is effective in preventing COVID-19 |
| 2 | Avoiding crowded places reduces the risk of contracting COVID-19 |
| 3 | If I practice physical distancing, I am less likely to get COVID-19 |
| **Perceived Response efficacy: Use of face masks** | |
| 4 | Face mask is effective in preventing COVID-19 |
| 5 | Wearing face mask regularly protects against COVID-19 |
| 6 | If I wear face mask regularly, I am less likely to contract COVID-19 |
| **Perceived Response efficacy: Hand hygiene practices** | |
| 7 | Cleaning hands regularly with soap and water or hand sanitizer is effective in preventing COVID-19 |
| 8 | Cleaning hands regularly with soap and water or hand sanitizer deters COVID-19 |
| 9 | I can reduce the risk of contracting COVID-19 by cleaning my hands regularly with soap and water or hand sanitizer |
| **Perceived Response efficacy: COVID-19 vaccination** | |
| 10 | COVID-19 vaccine protects against serious illness and death from COVID-19 |
| 11 | COVID-19 vaccine is effective in preventing serious COVID-19 disease or COVID-19 death |
| 12 | If I am vaccinated with COVID-19 vaccine, I will be protected from serious COVID-19 disease or COVID-19 death |

* Each item was assessed using a 5-point Likert scale ranging from 1 (strongly disagree) to 5 (strongly agree)
